# Exploring the relationship between cognitive abilities and motor performance in athletes with intellectual disabilities

**DOI:** 10.3389/fspor.2025.1601355

**Published:** 2025-06-30

**Authors:** Luca Cavaggioni, Damiano Formenti, Linda Casalini, Francesco Granito, Chiara Magro, Andrea Manente, Simona Canton, Nicola Lovecchio, Paolo Castiglioni, Giampiero Merati

**Affiliations:** ^1^ Department of Biotechnology and Life Sciences, University of Insubria, Varese, Italy; ^2^ Obesity Unit and Laboratory of Nutrition and Obesity Research, Department of Endocrine and Metabolic Diseases, IRCCS Istituto Auxologico Italiano, Milan, Italy; ^3^ Sciences of Preventive and Adapted Physical Activities, University of Insubria, Varese, Italy; ^4^ School of Medicine in Sports and Exercise, University of Insubria, Varese, Italy; ^5^ School of Medicine in Sports and Exercise, University of Verona, Verona, Italy; ^6^ Department of Human and Social Sciences, University of Bergamo, Bergamo, Italy; ^7^ IRCCS Fondazione Don Carlo Gnocchi Onlus, Milan, Italy

**Keywords:** intellectual disability, basketball, correlation analysis, fitness, intellectual and developmental disabilities

## Abstract

**Introduction:**

Basketball practice for athletes with intellectual disabilities (ID) is an ancient activity that stimulates cognitive and motor performance domains. This study aims to verify the association between cognitive performance and motor abilities in basketball athletes with ID.

**Methods:**

A total of 23 participants with ID were screened on cognition (clinical reaction time and Bells test 30 s and 90 s), motor performance [handgrip strength test (HST), countermovement jump (CMJ), static balance], and anthropometry [body mass index (BMI) and skinfolds] in a cross-sectional design.

**Results:**

A *strong* negative relationship was observed between clinical reaction time with HST and CMJ variables. A *strong* positive association was also found between Bells test 30 s with anthropometric variables (BMI) and power-related CMJ outcomes. Linear regression models revealed that the CMJ concentric mean force explained 34.3% of the variance of performance time during the clinical reaction time, and the HST combined with BMI explained 53% of the variance of cognitive ability during the Bells test 30 s.

**Conclusions:**

These results suggest a positive correlation between cognitive and motor performance in basketball players with ID. These findings encourage further exploration of how sports interventions could ameliorate physical and cognitive health in individuals with ID.

## 1 Introduction

Intellectual disability (ID) is a condition characterized by significant limitations in both intellectual functioning and adaptive behavior that originates before the age of 22 (1). Sports and physical activity are widely acknowledged as useful strategies to improve social, psychological, and physical well-being of individuals with ID (2). The practice of basketball for athletes with ID dates to 1994 in Greece with the first World Championship. Basketball is an open-skilled activity requiring short reaction times and decision-making process in relation to an unpredictable context (3) integrated with multiple physical, neuromuscular, technical, and psychological components. Notably, the nature of intermittent activities is quite complex (4), in particular for individuals with ID who tend to have a decline in neuromuscular and strength-related performance (i.e., muscular weakness, lower voluntary motor unit activation), cardiorespiratory fitness body composition, and technical skills compared with their typically developed peers (5–7). Moreover, players with ID present lower cognitive abilities compared with their able-bodied peers such as reaction time, decision-making, perception, execution process, and visuospatial attention (8). Specifically, visuospatial attention is the cognitive mechanism that enables the selective processing of visual information based on spatial location, enhancing perception and response to stimuli in the attended area (9). This skill presents a performance decay, especially when visual and spatial demands are combined (10, 11). Notably, the importance of cognitive abilities during open-skill team sports has been demonstrated in several-able-bodied research in basketball, volleyball, and football activities (12–14). To sum up, general cognitive functions are determinant components in team sports where perceptual and cognitive demands are expressed at high levels (13).

Previous studies have explored these associations showing that cumulative scores of cognitive abilities are positively associated with motor tests in soccer players (15). On the same line of evidence, Trecroci and colleagues found small-to-medium relationships between cognitive and motor skills in young volleyball players. The authors interpreted these associations as likely reflecting the complexity of motor skill assessments, which involve multiple abilities. This link may support improvements in both sport-specific and broader social and well-being domains (16). Nevertheless, when dealing with athletes with ID practicing an open-skilled activity as basketball, the combination between cognition and motor performance may offer a valuable perspective on the manner in which sport interventions could ameliorate physical and cognitive health status, thanks to the promotion of social inclusion, life skills development, and a feeling of accomplishment (17).

However, similar to previous evidence (12, 13, 15, 16), the present research intends to provide useful data to scientific community on the relationship between general cognitive function and motor performance in athletes with intellectual disabilities. Comprehending these connections would promote further debates on the potentiality of these connections as determinants of sport, especially for an open skill activity like basketball for individuals with ID. An accurate description of motor abilities of individuals with different types of disabilities (i.e., intellectual, motor or sensory impairments) is also crucial to develop individualized training sessions to enhance their functional capacities (18, 19).

Thus, the aim of the present study was to verify the association between cognitive performance (i.e., simple reaction time, visuospatial attention) with motor abilities (i.e., muscular fitness, balance) and body composition [i.e., body mass index (BMI), skinfolds] in basketball athletes with ID. Specifically, our hypothesis was that athletes with ID with quicker reaction times and greater visuospatial attention abilities would in parallel demonstrate higher neuromuscular-strength parameters together with better body composition outcomes.

## 2 Methods

### 2.1 Design setting

This retrospective, cross-sectional study was conducted in a single experimental session, lasting approximately 30 min, in the gym facilities close to the basketball court before the start of a basketball tournament for “Pro” and “Comp” categories (time of the day 10:00–15:00, temperature 20–23°C). In this regard, the Italian Sport Federation for Athletes with Intellectual and Relationship Disability (FISDIR) proposes three basketball levels: promotional (“Pro”), middle-regional (“Comp”), and national (“Open”).

### 2.2 Participants

Thirty-three athletes with ID voluntarily participated in this study and were screened for preliminary eligibility. A sample of 33 participants playing within different basketball teams in the “Pro” and “Comp” category were recruited (age: 29.1 ± 8.8 years, height: 1.72 ± 0.10 m, body mass: 80.2 ± 20.5 kg, BMI: 27.1 ± 6.9 kg/m^2^, m ± SD, male/female: 24/9, level of intellectual disability: mild-to-moderate). For the present research, the inclusion criteria were (i) age between 18 and 30 years, and (ii) recognized ability to understand verbal communication. The exclusion criteria were: (i) presence of any clinical, physical, or mental condition that may compromise the regular practice of basketball activity or the inability to perform all testing procedures in a correct manner; (ii) strict dependence from personnel or assistive support devices during testing procedures; and (iii) regular basketball practice with a minimum frequency of two training sessions per week. After a specified explanation of the study's aims, risks, and benefits, athletes or their legal guardians signed the written informed consent to participate. The research complies with the Helsinki Declaration on studies with human participants and was approved by the local University Ethics Committee (protocol number 0035482, date 12/03/2024).

### 2.3 Data collection

Cognitive performance was assessed using the clinical reaction time (20) and the Bells test (21).

The clinical reaction time test detects the simple reaction time ability and was performed following previously described procedures (22). In summary, the athletes were instructed to catch an 80-cm wooden dowel coated in high-friction tape and marked in 0.5-cm increments as quickly as possible. The dowel was embedded in a weighted rubber disk (diameter = 7.5 cm, height = 2.5 cm, and mass = 256 g). Athletes sat on a chair with the dominant hand positioned at the edge of the table in an open C-shape position with their fingers. The examiner initially held vertically the dowel so that the weighted rubber disk was in line with the participant's first and second digits. Subsequently, he released down the dowel at a random time interval (from 4 to 10 s) to prevent from anticipating the time of release. Participants should have caught the dowel as quickly as possible with their dominant hand while maintaining gaze on the weighted disc. The distance in centimeters covered from the top of the disk to the most superior part of the dowel's marked increments was recorded. The reaction time value was calculated by converting distance to time, in milliseconds, using the formula for a body falling under the influence of gravity (D = 1/2 g t^2^). The test was administered by the same operator for all athletes. After two practice trials, participants performed four experimental trials, and the mean value was considered for the analysis. This testing procedure shows excellent test–retest (ICC = 0.86) and inter-rater reliability (ICC = 0.74) in healthy individuals (23). Nevertheless, reliability coefficients have not yet been investigated in individuals with ID and this could be a potential limitation. The Bells test is a neurocognitive assessment able to measure visuospatial attentional pattern (21). It consists of the participant's ability to cross out with a pencil all the 35 bells' figures that are drawn and scattered among several distractors' shapes (houses, horses, etc.) on a A4 sheet of paper placed exactly in front of them and aligned with their mid-sagittal plane. The task was considered finished when the athlete stated to have completed the required task or at the end of the fixed time of 30 s and 90 s. The total number of circled bells was recorded in the two conditions: 30 s and 90 s. While this test has a validity in individuals with a stroke (24), its reliability and internal consistency in participants with cognitive impairments needs to be further investigated. This may underscore a potential bias acknowledged in the limitations section. With regard to motor performance, the Handgrip Strength Test (HST) and the countermovement jump performance were employed to assess neuromuscular components in terms of maximal isometric and explosive strength qualities. HST is a valid and reliable indicator regarding whole-muscle strength (25). It was performed with a digital dynamometer (Camry EH101, Sensun Weighing Apparatus Group Ltd, Guangdong, China) widely used within different populations (26). The grip strength was detected three times for each hand, starting with the dominant one, with the participant sitting on a chair with his elbow flexed at a 90° angle. Participants were requested to maximally squeeze the dynamometer for three consecutive seconds. Strength asymmetry was calculated as the ratio between handgrip strengths of the nondominant and dominant hand, in percentage. Three trials were given (separated by 1 in of rest), and the mean value was considered for the statistical analysis. Previous evidence shows a higher reliability coefficient (ICC = 0.96) in individuals with intellectual disabilities (27).

The countermovement jump (CMJ) was used to assess explosive strength by jumping over two uniaxial portable force plates (ForceDecks, FDLite V.2, VALD, Brisbane, Australia) (28). A stacked force plate arrangement enabled the measurement of vertical ground reaction forces and center of pressure simultaneously. Both force plates recorded data at 1,000 Hz and were connected to a computer through the official software (VALD ForceDecks software for Windows, V2.0.8587) to analyze the signal and to return the most common neuromuscular jumping variables (i.e., jump height in centimeters, peak power in Watt, maximum concentric rate of force development in Newton per seconds, concentric and eccentric mean force in Newton, peak landing force in Newton, concentric peak velocity in meter per seconds, and the modified Reactive Strength Index, in meter per seconds by dividing the jumping height with the contraction time). Before starting the jumping action, during the preparatory phase, each athlete was instructed to bend his knees faster and to jump as high as possible. Three trials were conducted observing 3-min rest in between. This assessment has been shown to have reliability coefficients with Cronbach's alpha exceeding 0.75 in participants with ID (29).

Static balance was determined with athletes in upright, quiet, barefoot, bipedal stance over two portable force plates (ForceDecks, FDLite V.2, VALD, Brisbane, Australia) with eyes closed and arms at their sides, remaining in the proper position as long as they could until a maximum of 30 s. Both ForceDecks plates recorded the center of pressure displacement (COP) at 200 Hz. Three trials were performed while respecting an interval rest of 1 min. VALD ForceDecks software for Windows (V2.0.8587) analyzed COP raw signals and the following parameters were considered for statistical analysis: antero-posterior and medio-lateral COP sway area in millimeters squared and COP mean velocity in millimeters per seconds. This testing procedure presents ICC reliability coefficients ≥0.70 in individuals with ID (30).

For what concerns anthropometry, body mass was determined using a SECA 877 scale (SECA® 877, Hamburg, Germany) to the nearest 100 g, body height in meters was measured to the nearest 0.1 m adopting a SECA 217 vertical stadiometer (SECA® 240, Hamburg, Germany), and BMI was calculated in kilograms per meters squared. Body composition was determined by skinfold measurements, estimating body fat from several folds of skin thickness across various body regions using a plicometer. The assumption is that the amount of subcutaneous fat is proportional to the total amount of body fat while considering interindividual variations (31). In detail, biceps, triceps, subscapular, and medial calf sites were used respecting the procedures suggested by the American College of Sports Medicine international guidelines (31).

### 2.4 Statistical analysis

All data are presented as mean ± standard deviation (SD). The dataset has been tested for normal distribution using Shapiro–Wilk's normality test. Pearson correlation coefficient (r) or in case of non-normality distribution, Spearman correlation coefficient (ρ) was calculated to provide associations between cognition and motor variables. In addition, a stepwise multiple regression was performed to predict cognitive performance. Specifically, motor performance that most significantly correlated with clinical reaction time and Bells tests were entered into the stepwise procedure. Correlation coefficients of 0.1, 0.3, 0.5, 0.7, and 0.9 were considered s*mall, moderate, large, very large,* and *extremely large* (32). The effect size interpretation was 0–0.3 *weak* effect, 0.3–0.5 *moderate* effect, and more than 0.5 *strong* effect (33). With regard to sample size determination, it was based on data of performance in one primary outcome (i.e., handgrip strength test) conducted in a previous study on a sample of individuals with ID (34) using G*Power software. From *a priori* power analysis, it is required to recruit a minimum sample of 30 individuals (*β* = 0.80) respecting a large effect size (Cohen's d equal to 0.4) with a type I error rate of *α* = 0.05. All statistical analyses were performed using the Statistical Package for Social Sciences, IBM™ SPSS™ Statistics (version 21.0, IBM Corp., Somers, Chicago, IL, USA).

## 3 Results

**Figure 1:**
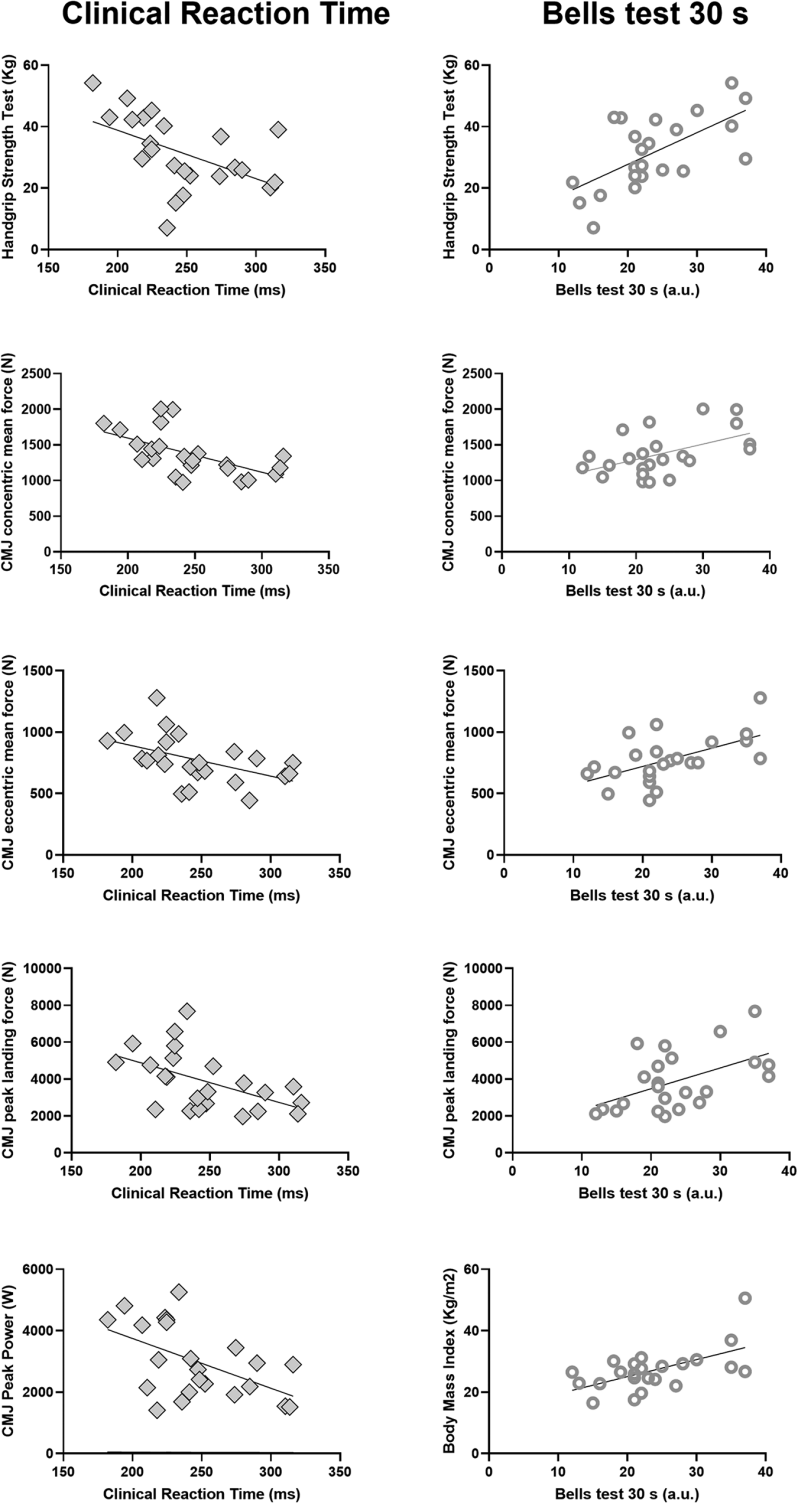
Correlation between clinical reaction time (left panels) or bells test score (right panels) with various physical test metrics. Only variables with statistically significant (*p* < 0.05) linear correlation are reported. Each plot shows individual data points and the regression line (see correlation coefficients and significance levels in Table 1).

Starting from the initial 33 athletes, 10 of them were excluded because they did not meet the exclusion criteria, so a final sample of 23 participants (age: 29.1 ± 8.8 years, height: 1.72 ± 0.10 m, body mass: 80.2 ± 20.5 kg, BMI: 27.1 ± 6.9 kg/m^2^, m ± SD) was considered. Because of the low sample size, for analyses convenience, athletes belonging to “Pro” and “Comp” categories were unified in a unique group. The mean ± SD value for the clinical reaction time test was 246.4 ± 37.8 ms. With regard to the Bells test, the mean ± SD values of the 30 s trial was 23.7 ± 7.3 a.u. and 37.3 ± 5.8 a.u. for the 90 s trial, respectively. Descriptive statistics of motor performance, anthropometry, and body composition are presented in Table 1, together with their correlation coefficients with cognitive tests. A significant *large* negative relationship was found between clinical reaction time performance and handgrip strength test (*r* = −0.50, *p* = 0.014, *strong* effect), peak power output during a CMJ (*r* = −0.52, *p* = 0.011, *strong* effect), a CMJ concentric mean force (*r* = −0.58, *p* = 0.003, *strong* effect), CMJ eccentric mean force (*r* = −0.48, *p* = 0.019, *moderate* effect), and CMJ peak landing force (*r* = −0.51, *p* = 0.012, *strong* effect) parameters. Static balance and body composition outcomes presented no significant relationships (Figure 1).

**Table 1:** Participants’ main characteristics and correlation coefficients with cognitive tests.

Variables	Mean ± SD	Correlation coefficient (r) or (ρ) with		
		Clinical reaction time (ms)	Bells test 30 s (a.u.)	Bells test 90 s (a.u.)
Clinical reaction time (ms)	246.4 ± 37.8	–	–	–
Bells test 30 s (a.u.)	23.7 ± 7.3	–	–	–
Bells test 90 s (a.u.)	37.3 ± 5.8	–	–	–
Handgrip strength test (kg)	31.5 ± 11.8	−0.50*	0.63**	−0.01
Handgrip strength test asymmetry (%)	14.2 ± 15.6	−0.20	0.17	−0.15
CMJ jump height (cm)	22 ± 11.2	−0.25	0.23	0.01
CMJ peak power (W)	2,997.3 ± 1,175.6	−0.52*	0.35	0.11
CMJ maximum concentric rate of force development (N/s)	4,616.8 ± 2,457.3	0.23	−0.17	0.20
CMJ concentric mean force (N)	1,374.3 ± 309.3	−0.58**	0.50*	−0.04
CMJ eccentric mean force (N)	774.9 ± 193.7	−0.48*	0.55**	−0.02
CMJ peak landing force (N)	3,886.1 ± 1,580	−0.51*	0.51*	−0.07
CMJ concentric peak velocity (m/s)	2.1 ± 0.5	−0.32	0.14	0.18
Modified reactive strength index (m/s)	0.3 ± 0.1	0.34	0.13	−0.27
Antero-posterior COP sway area (mm^2^)	87 ± 243.7	−0.28	0.10	−0.11
Medio-lateral COP sway aerea (mm^2^)	46.7 ± 35.4	−0.35	0.07	0.11
COP mean velocity (mm/s)	13.1 ± 5.1	−0.11	−0.06	0.19
Body mass index (kg/m^2^)	27.1 ± 6.9	−030	0.48*	−0.09
Bicep skinfold (mm)	7.8 ± 4.9	0.12	0.04	0.27
Tricep skinfold (mm)	13 ± 5.5	0.08	0.03	−0.02
Subscapular skinfold (mm)	19.3 ± 8.6	0.00	0.12	−0.13
Medial calf skinfold (mm)	11.2 ± 5.5	−0.12	0.40	−0.28

SD, standard deviation.

* *p* < 0.05.

** *p* < 0.01.

For what concerns the Bells test 30 s, a *large* positive association was revealed for the handgrip strength test (*r* = 0.63, *p* = 0.001, *strong* effect), body mass index (*ρ* = 0.48, *p* = 0.003, *moderate* effect), CMJ concentric and eccentric mean force (*r* = 0.50, *p* = 0.013, *moderate* effect; *r* = 0.55, *p* = 0.006, *strong* effect), and CMJ peak landing force (*r* = 0.51, *p* = 0.011, *strong* effect) (Figure 1). No significant associations were detected between measures of neuromuscular performance assessed through the CMJ or the handgrip strength test, static balance, and body composition variables with Bells test 90 s value.

**Figure 2:**
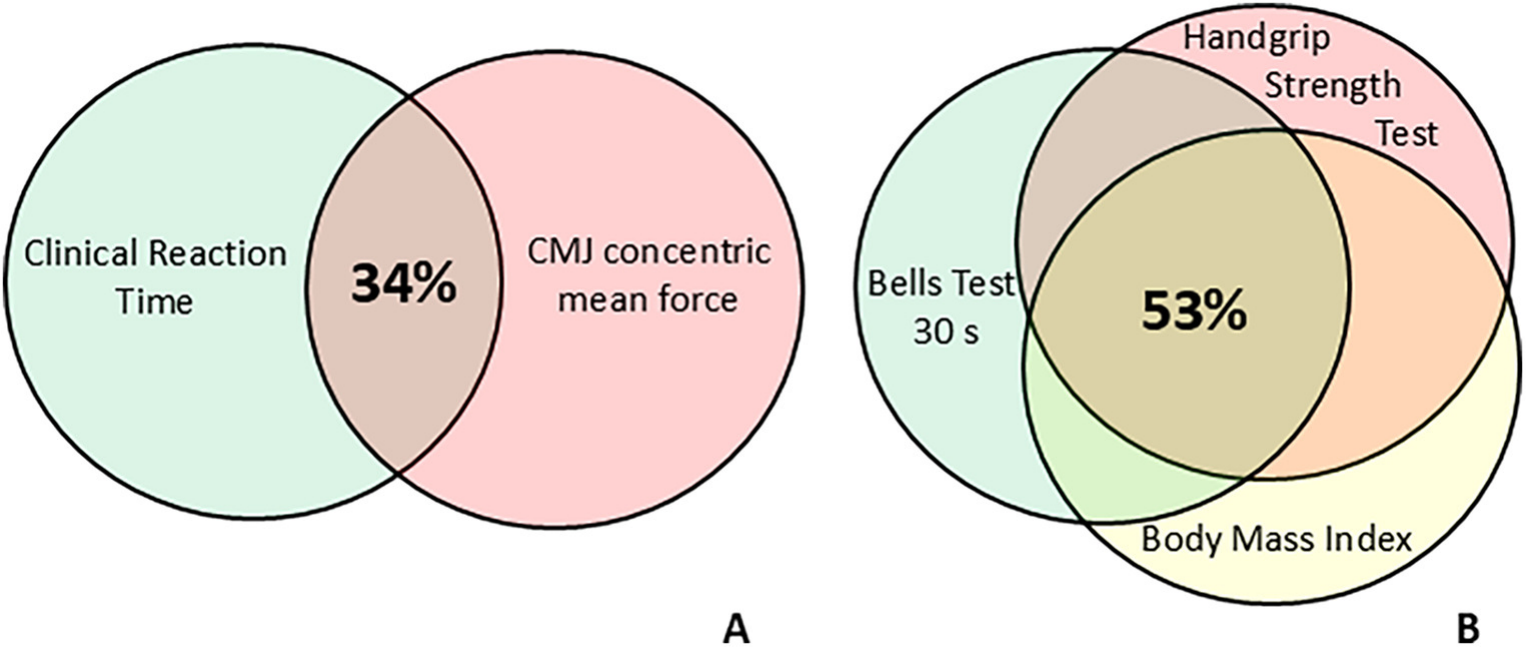
Venn diagrams representing the percentage of explained variance estimated as adjusted R^2^ by predictors of clinical reaction time **(A)** and Bells test 30 s **(B)** after stepwise multiple regression. The overlap in Diagram A shows that the CMJ concentric mean force explains more than one-third of the clinical reaction time variance. The overlap in Diagram B shows that the handgrip strength test and body mass index independently explain more than half of the “Bells test 30 s” variance.

Table 2 highlights the results derived by the linear regression analysis. In detail, the stepwise multiple linear regression analysis with clinical reaction time as a dependent variable showed that the CMJ concentric mean force as predictor accounted for 34.3% (*p* = 0.003) (adjusted R^2^) in explaining the result of time in seconds performance variance (Figure 2A). Likewise, for Bells test 30 s, the stepwise multiple regression highlighted that handgrip strength test and body mass index parameters were predictors that both are able to explain for the 53% (*p* = 0.0011) the total number of circled bells result (dependent variable) of the model variance (adjusted R^2^) (Figure 2B).

**Table 2:** Linear regression models for cognitive motor performances.

Independent predictors	R	R^2^	Adjusted R^2^	Std. error of the estimate	R^2^ change	F change	Beta	df1	df2	Sig. F change
Clinical reaction time predictors	0.586	0.448	0.343	31.377	0.343	11.004	−0.586	1	21	0.003
CMJ concentric mean force										
BMI, HST	0.757	0.572	0.530	4.976	0.169	7.932	0.503	1	21	0.010

CMJ, countermovement jump; BMI, body mass index; HST, handgrip strength test.

## 4 Discussion

The purpose of the study was to investigate the association between cognitive and motor performance in basketball athletes with ID. The first main finding was that a *large* negative significant relationship was observed between clinical reaction time and upper and lower-body neuromuscular performances (i.e., handgrip strength test and countermovement jump variables), suggesting that the higher the muscle strength, the lower the reaction time. The second main finding was also a *large* positive significant association between Bells test 30 s with anthropometric variables (i.e., BMI) and power-related CMJ outcomes, indicating that a higher neuromuscular performance during a CMJ or HST is accompanied by a better visuospatial attention. The third main finding was that linear regression models revealed that muscle-strength related outcomes are able to determine the relationship between motor abilities and cognitive performance in athletes with ID practicing basketball. Overall, this study demonstrated that ID basketball athletes with superior cognitive performance (expressed by clinical reaction time and Bell test score) presented also better motor performance (expressed by strength-related outcomes). In detail, the concentric mean force expressed during a CMJ explained 34.4% of the performance time variance obtained during the clinical reaction time test. Moreover, the handgrip strength test associated with body mass index explains for 57.3% the athletes' cognitive ability to recognize the maximum number of bells during the Bells test 30 s. Research has demonstrated that individuals with ID display a lower muscular fitness and power-related variables especially in lower-limb segments. Specifically, ID athletes jump lower as compared to their typically developed peers and this result may be attributed to a reduced motor unit activation and electromyographic activity (6). When analyzing the CMJ pattern, the present athletes with ID display a reduced peak power output, take-off velocity, and lower-limb muscle tendon unit stiffness with an inefficient stretch-shortening cycle mechanism (29). In fact, all these aspects are crucial in determining an effective vertical jumping performance (35). Lastly, when dealing with upper-body strength, cumulative evidence observed a reduced handgrip performance in participants with ID due to a reduced manipulation ability and quick reactions to hold and carry objects (36).

When it comes to basketball performance, research has documented the importance of exerting higher levels of muscular power and force to perform maximal jumps, sprints, change of directions or the mastery to use hands during catching, holding, shooting or throwing the ball (37). In addition, basketball is also influenced by several cognitive abilities such as selective spatial attention, general intelligence, perceptive analysis, and logical conclusions (38). Players have to perceive the court situation and choose the optimal motor action, in accordance with tactical situations during unexpected game situations (39). Athletes with ID show a lower reaction time, decision-making, perception, attention, and execution process during a basketball game (40, 41) with a concomitant delay in technical skills proficiency (7). The quantification of cognitive performance and the identification of its correlation with other variables, such as motor performance, body composition may help to support objective parameters for the indications in this sport to optimize physical activity interventions for individuals with ID. Besides improving the understanding of these interactions, our results could contribute to depict a more accurate picture regarding athletes' abilities and may serve as a basis for more effective, tailored preventive measures for individuals with intellectual disabilities. Lastly, from a speculative point of view, the comprehension of the associations may offer future perspectives in helping the classification process to become even more accurate by focusing not only on athletes' cognitive level but also on their functional capacities having an influence on basketball performance for athletes with ID.

Future studies should be geared to investigate this scenario with the aim of obtaining new findings related to a more independent and fulfilling quality of life in a larger cohort of individuals with ID. Moreover, given the heterogeneity of syndromes encompassed within intellectual disabilities (e.g., Down syndrome, Rett syndrome, Williams syndrome, Autism Spectrum Disorders), each characterized by distinct clinical, physiological, and sport-specific technical profiles, it would be valuable to further investigate the association we identified while differentiating between each syndrome, competitive level, or technical abilities. Nevertheless, there are several limitations that should be acknowledged. First, the low sample size and non-distinction between various ID syndromes limits the possibility to generalize our result to other cognitive-impaired conditions. Second, the lack of other physiological parameters (e.g., heart rate, lactate blood sample) or technical skills reduces the possibility to reinforce the associations between parameters (42). Third, the lack of comparison between basketball athletes with ID within different competitive levels (“Pro” Vs “Comp”, Vs “Open”) brings caution to data interpretation. Fourth, the lack of established reliability and validity of the clinical reaction time and Bells test for individuals with intellectual disabilities (ID) represents a potential limitation and source of bias, warranting cautious interpretation of the main findings.

## 5 Conclusion

This study revealed a significant relationship between cognitive and motor performance in athletes with ID athletes. These associations are particularly remarkable between neuromuscular and strength-related outcomes, clinical reaction time, and Bells test scores. Although this is only an initial attempt to understand the relationship between cognitive and motor behaviors in athletes with ID, these findings highlight the importance of expanding the current knowledge derived from able-bodied athletes to explore the relationships between cognitive and motor skills within a sports performance context in the population with ID. Comprehending these connections could foster further discussions on their potential role as determinants not only of sport performance but also of health status in individuals with ID.

## Data Availability

The raw data supporting the conclusions of this article will be made available by the authors without undue reservation.
