# Supplementary material for: Exploring the relationship between cognitive abilities and motor performance in athletes with intellectual disabilities
Source: Front Sports Act Living. 2025 Jun 30;7:1601355. doi: 10.3389/fspor.2025.1601355 (PMC12256452; doi:10.3389/fspor.2025.1601355)
Supplement: Supplementary file 1 [file Datasheet1.pdf]

Azioni rapide | Già letto/Da leggere

Tutto | Posta | File | Teams | Persone | Contiene allegati

Risultati principali

Formenti Damiano
analisi Valmadrera — 14/11/2024
Ciao Lu, ti giro anche quel poco che avevo iniziato a scriv... — Posta in arrivo
Paper Valmadre...

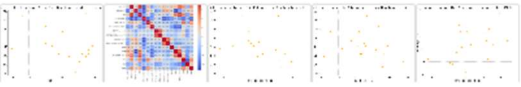

Merati Giampiero
Paper valmadrera basket — 31/12/2024
Caro Luca, grazie mille per il tuo lavoro. Purtroppo non ri... — Posta in arrivo

104

Cavaggioni Luca
Paper valmadrera — 23/12/2024
Ciao Dami! ti mando il paper di valmadrera in google driv... — Posta inviata
Paper Valmadre... | Paper Valmadre... | Paper Valmadre...

4

Paper dati Valmadrera

Cavaggioni Luca
A: Merati Giampiero; Formenti Damiano; — mer 29/01/2025 15:15
Paolo CASTIGLIONI <paolo.castiglioni@gmail.co...
Linda Casalini <casalinilinda@gmail.com>;
Linda Casalini <linda.casalini@outlook.it>;
CANTON SIMONA; MANENTE ANDREA;
nicola.lovecchio@unibg.it

Cari prof,

vi mando il paper sui dati raccolti coi ragazzi con DS a Valmadrera (ringrazio i prof. Merati e Formenti per il coinvolgimento).

Vorremmo mandarlo sulla rivista SPORTS di mdpi (IF 2.2 - Q1 per le aree tematiche "sports therapy and rehabilitation" e "physical therapy).

Vi invio l'articolo con googledrive così è più comodo revisionare il testo:

https://docs.google.com/document/d/16hIFkz5d_RCfwsf5QRZQv0ggIt0HlvQFaLMgP8-RAVU/edit?

paper — Posta in arrivo ×

Nicola LOVECCHIO <nicola.lovecchio@unibg.it> — 31 gen 2025, 18:00
a me, Formenti

Grazie Luca
hai fatto un grande lavoro.
nel file condiviso ho messo i miei commenti (in arancione)
ho aggiunto una frase nella conclusione (in arancione e in italiano) che se volete accogliere lascia uno spazio di riflessione come practical implication.
